# Developing a standardized approach to the assessment of pain in children and youth presenting to pediatric rheumatology providers: a Delphi survey and consensus conference process followed by feasibility testing

**DOI:** 10.1186/1546-0096-10-7

**Published:** 2012-04-10

**Authors:** Jennifer N Stinson, Mark Connelly, Lindsay A Jibb, Laura E Schanberg, Gary Walco, Lynn R Spiegel, Shirley ML Tse, Elizabeth C Chalom, Peter Chira, Michael Rapoff

**Affiliations:** 1 University of Toronto Lawrence S. Bloomberg Faculty of Nursing, 155 College Street, Toronto ON M5T 1P8, Canada; 2 University of Toronto, Department of Pediatrics, 1 King's College Circle, Toronto ON M5S 1A8, Canada; 3 The Hospital for Sick Children, Child Health Evaluative Sciences, 555 University Avenue, Toronto ON M5G 1X8, Canada; 4 The Hospital for Sick Children, Department of Anesthesia and Pain Medicine, 555 University Avenue, Toronto ON M5G 1X8, Canada; 5 The Hospital for Sick Children, Department of Pediatrics, 555 University Avenue, Toronto ON M5G 1X8, Canada; 6 Children's Mercy Hospitals and Clinics, Pain Management Program, 2401 Gillham Road, Kansas City MO 64108, USA; 7 Duke Medical Center, Department of Pediatrics, DUMC 3212, Durham NC 27710, USA; 8 Seattle Children's Hospital, Department of Anesthesia and Pain Medicine, 4800 Sand Point, Way NE Seattle WA 98105, USA; 9 Saint Barnabas Medical Center, Department of Pediatrics, 94 Old Shore Hills Road, Livingston NJ 07039, USA; 10 Indiana University School of Medicine, Department of Pediatrics, 705 Riley Hospital Drive, Indianapolis IN 46202, USA; 11 University of Kansas Medical Center, Department of Pediatrics, 3901 Rainbow Boulevard, Kansas City KS 66160, USA

**Keywords:** Pain, Rheumatic diseases, Pediatrics, Feasibility studies, Pain measurement

## Abstract

**Background:**

Pain in children with rheumatic conditions such as arthritis is common. However, there is currently no standardized method for the assessment of this pain in children presenting to pediatric rheumatologists. A more consistent and comprehensive approach is needed to effectively assess, treat and monitor pain outcomes in the pediatric rheumatology population. The objectives of this study were to: (a) develop consensus regarding a standardized pain assessment tool for use in pediatric rheumatology practice and (b) test the feasibility of three mediums (paper, laptop, and handheld-based applications) for administration.

**Methods:**

In Phase 1, a 2-stage Delphi technique (pediatric rheumatologists and allied professionals) and consensus meeting (pediatric pain and rheumatology experts) were used to develop the self- and proxy-report pain measures. In Phase 2, 24 children aged 4-7 years (and their parents), and 77 youth, aged 8-18 years, with pain, were recruited during routine rheumatology clinic appointments and completed the pain measure using each medium (order randomly assigned). The participant's rheumatologist received a summary report prior to clinical assessment. Satisfaction surveys were completed by all participants. Descriptive statistics were used to describe the participant characteristics using means and standard deviations (for continuous variables) and frequencies and proportions (for categorical variables)

**Results:**

Completing the measure using the handheld device took significantly longer for youth (M = 5.90 minutes) and parents (M = 7.00 minutes) compared to paper (M = 3.08 and 2.28 minutes respectively p = 0.001) and computer (M = 3.40 and 4.00 minutes respectively; p < 0.001). There was no difference in the number of missed responses between mediums for children or parents. For youth, the number of missed responses varied across mediums (p = 0.047) with the greatest number of missed responses occurring with the handheld device. Most children preferred the computer (65%, p = 0.008) and youth reported no preference between mediums (p = 0.307). Most physicians (60%) would recommend the computer summary over the paper questionnaire to a colleague.

**Conclusions:**

It is clinically feasible to implement a newly developed consensus-driven pain measure in pediatric rheumatology clinics using electronic or paper administration. Computer-based administration was most efficient for most users, but the medium employed in practice may depend on child age and economic and administrative factors.

## Background

Pain in children and youth with rheumatic conditions such as arthritis is common [1,2]. Currently, there is no standardized approach guiding the clinical assessment of pain in children and youth presenting to pediatric rheumatologists and other allied health professionals. Consequently, large variations exist across rheumatology practices in the evaluation and treatment of pediatric pain. It has recently been recommended that pain assessment, along with other quality measures, should be routinely implemented in pediatric rheumatology practices as a means of tracking outcomes and generating quality improvement indices [3]. Thus, a consistent, comprehensive, and clinically feasible approach is needed to effectively assess, treat, and monitor pain outcomes in the pediatric rheumatology population.

As pain is a multi-dimensional experience, appropriate pain assessment requires evaluation of sensory, affective and cognitive dimensions and the impact of pain on aspects of a child's life. The Pediatric Initiative on Methods, Measurement, and Pain Assessment in Clinical Trials (PedIMMPACT) recently completed consensus guidelines for dimensions of pain to assess as outcomes in clinical trials [4]. These recommendations offer a foundation for evaluating the key components of pain to be integrated into clinical assessment, including pain intensity, global rating of satisfaction with pain treatment(s) received, additional symptoms and adverse events, physical functioning, emotional functioning, role functioning, sleep, and economic factors. However, no consensus guidelines exist for the assessment of these multiple dimensions of pain in routine rheumatology practice.

The ultimate aim of this study was to develop and test the feasibility of a Standardized Universal Pain Evaluation for pediatric rheumatology providers (SUPER-KIDZ). This aim was accomplished using a 2-phased approach. First, consensus was sought from rheumatologists and pediatric pain experts on the most important pain domains to assess during routine clinical rheumatology practice, using the domains recommended by PedIMMPACT as a preliminary guide. Second, feasibility was evaluated by administering the pain assessment tool in three formats (paper-, laptop-, and handheld computer-based applications) and determining acceptability, efficiency, and missed responses in four large pediatric rheumatology clinics. In the present study, we elected to evaluate medium feasibility before conducting validity testing as test validity has previously been shown to be sensitive to the test medium employed [5].

## Methods

### Phase 1: Developing consensus on the SUPER-KIDZ Pain Measure

A 2-stage Delphi technique was used to develop consensus amongst Childhood Arthritis and Rheumatology Research Alliance (CARRA) members (pediatric rheumatologists and other allied professionals) as to what aspects of pain assessment to include in the evaluation of children and youth presenting to rheumatology clinics. For purpose of this study, the word 'child' or 'children' refers to persons aged 4-7 years and the word 'youth' refers to persons aged 8-18 years. The Delphi method was originally developed by the RAND Corporation in the 1950s to obtain reliable consensus of opinion from a group of experts through a series of questionnaires interspersed with controlled opinion feedback [6]. This technique was recently used to develop consensus on the predictive factors of pediatric chronic pain and pain-related disability [7] and has been used successfully by CARRA, the Pediatric Rheumatology International Trials Organization (PRINTO), the Pediatric Rheumatology Collaborative Study Group (PRCSG) and the American College of Rheumatology (ACR) in the past [3,8-13]. A two day consensus conference followed, with the goal of reaching a final consensus on the domains and items to be measured using SUPER-KIDZ.

#### Delphi Survey - Iteration 1

Following approval from the CARRA Pain Disease Specific Group and Steering Committee, an e-mail (with two subsequent reminder e-mails over a 3 week period) was sent to all CARRA members inviting their participation in a survey regarding the development of a uniform pain assessment for pediatric rheumatology clinics. Potential respondents were informed of the study purpose and participation requirements for the first iteration of the Delphi procedure and directed to the electronic survey site (Survey Monkey). Respondents rated the importance of the domains recommended by PedIMMPACT for inclusion in the pain assessment of children and youth presenting to rheumatology clinics using a 0 - 10 scale (0 = not at all important and 10 = extremely important) and generated any additional domains deemed important. Respondents were asked to give separate ratings of importance based on age group of the participant being assessed (ages 4-7 versus ages 8-18) and presumed diagnosis (arthritis, idiopathic musculoskeletal pain, or other rheumatic conditions). Respondents were also asked if and how their rating would change at different points of assessment (initial versus follow-up). Respondents gave importance ratings for each domain by every combination of time point, age and diagnosis. This resulted respondents answering 96 questions for Iteration 1.

#### Delphi Survey - Iteration 2

In the second iteration of the Delphi procedure, another e-mail was sent to all CARRA members as was done in the first iteration. Interested respondents then rated the importance of items (using the same 0 - 10 scale) within each assessment domain (generated from the first iteration) and then for each overall domain. Respondents also were asked to indicate whether and how (e.g. more important for youths than children) ratings would significantly increase or decrease based on the age or diagnosis of the patient. Because respondents were only prompted to indicate how their rating would change if they answered affirmatively to whether or not it would change, a range of questions (74-168) could be answered in Iteration 2.

#### Consensus Conference

A two-day consensus conference was held in May 2009 in Toronto, Canada. The meeting was attended by 8 pediatric pain experts of varying disciplines, 6 pediatric rheumatologists, 3 consumers, and 3 allied health professionals (physical and occupational therapists) from Canada and the United States. The overall goal of the meeting was to reach consensus on the pain domains and sub-domain items based on Delphi survey results based on age group of the participant being assessed. The meeting was facilitated by Drs. McGrath (PedIMMPACT meeting chair) and Walco (chair of the CARRA pain committee) using nominal group technique. Briefly, nominal group technique is a structured face-to-face meeting, with 2 round-robin voting guided discussions to facilitate reaching consensus among experts [10]. Attendees were provided with a brief synopsis regarding the goals of the conference and the technique that would be used to establish consensus. Each participant then had 1-2 uninterrupted minutes to speak to the group about which domains and sub-domains they felt were most relevant for inclusion in SUPER-KIDZ. Voting on the domains and sub-domains by all attendees then followed. Results from the vote were presented to attendees followed by a second opportunity to speak to the group regarding important domains and sub-domains. A second round of voting again followed. If a domain was endorsed by greater than 75% of participants, it was retained and the remainder were discarded.

### Phase 2: Feasibility Testing of Three Methods of Administering the Pain Measures

#### Participant Selection

Participants were recruited from four large CARRA member rheumatology clinics in university affiliated pediatric tertiary care centers across North America. Children and youth were eligible to participate if they were: (a) between 4-18 years of age; (b) diagnosed with or being assessed for a rheumatic condition by a rheumatologist; and, (c) English-speaking. Parents were eligible to participate if they: (a) had a child that met the study inclusion criteria; and (b) were English-speaking. All participants were excluded if they had major cognitive impairments. Study sample size was based on numbers thought to be realistically attainable within the study timeframe and which would provide adequate representation of the population of interest. No formal sample size determination was conducted as this was a pilot feasibility study with no specific hypotheses. The study was approved by Institutional Review Boards at all sites.

#### Procedures

Prior to their rheumatology appointment, eligible children (and parents) and youth were approached by a health care team member, and those interested in participating met with a research assistant (RA) who obtained informed consent. Demographic and disease characteristics were then obtained from participants and medical chart review, and a secure patient account was created on the SUPER-KIDZ website. Children and their parents and youth were given brief training (requiring less than 5 minutes *per *participant) on the nature of the assessment (i.e. rheumatic pain), the functionality of the electronic devices and how to complete the assessments using each of the paper, handheld device and laptop computer mediums. Participants then completed the same SUPER-KIDZ pain assessment using each of the three mediums. In all cases the RA was present in the room as surveys were completed and ensured that the assessments were completed by participants themselves, without the aid of others (i.e. family members). During this process, RAs recorded time to complete each survey. The sequence in which each medium was completed was randomly assigned using an Internet-based randomization scheme generator. The paper version of the pain assessment and a concise summary report from the laptop computer were provided to physicians prior to their clinical assessment. The computer summary report included composite scores related to the affective and evaluative domains of pain and therefore has fewer items to review. Following the visit, youth, parents and physicians completed a questionnaire related to their satisfaction with each pain assessment method. Physicians also provided a global disease severity rating for each participant immediately following the visit.

Both electronic mediums for each age group were programmed to present questions in chronological order with one question displayed on the screen at a time. After answering a question, the participant selected the word 'next' on the screen to proceed. If the question was not answered and the participant selected 'next', the program would present the same question for completion. If the question was again not answered and the participant selected 'next', the program would proceed to the following screen. Participants were unable to access a previous question once they had proceeded. These features were included to minimize the number of missed responses. The handheld device used was an iPod Touch (second generation) developed by Apple Incorporated with an 8 GB flash-drive and a 3.5" (diagonal) multi-touch display. Each site used their own laptop computer with Internet Explorer 7.0. Both devices accessed the Internet through local Wi-Fi services. The paper questionnaires presented multiple questions per page and participants were neither encouraged nor discouraged to review or modify responses.

#### Data Analyses

The quantitative data from the questionnaires were coded, scored and entered into a Statistical Package for the Social Sciences (SPSS) database [14]. Descriptive statistics were used to describe the participant characteristics using means and standard deviations (for continuous variables) and frequencies and proportions (for categorical variables). Efficiency and missed response data were analysed using Friedman's tests of analysis of variance for non-parametric data for a repeated-measures design. Acceptability data were analysed using χ^2^-tests. Differences were considered statistically significant at p ≤ 0.05 unless otherwise indicated.

## Results

### Phase 1: Developing consensus on the SUPER-KIDZ Pain Measure

#### Delphi Survey - Iteration 1

Of the 251 CARRA members invited to participate, 115 (46%) completed the survey. Participants were predominantly female (61%) and physicians (97%), with fellows comprising 16% of the physician group. Table 1 presents mean (M) and standard deviation (SD) values for importance ratings for pain domains by diagnosis and age grouping (time point was excluded from the Iteration 2 questions). The majority of respondents rated most domains as highly important to evaluate (overall M ± SD: 7.55 ± 1.44). Appointment type (i.e. initial or follow-up) did not generally affect importance ratings, and "satisfaction with treatments received" was viewed by respondents as being most relevant at follow-up (M = 7.4 versus 6.7); *t*(72) = 3.85, p < 0.01). Respondents tended to rate domains as significantly more important to assess in youth versus children, with the exception of physical functioning which was rated equally important for both age groups. Importance ratings also differed on domains contingent on the nature of the presenting problem: importance of assessing symptoms and adverse effects was rated significantly lowest, and importance of assessing emotional functioning and sleep was rated significantly highest for patients with presumed idiopathic musculoskeletal pain. Free marginal kappas [15,16] ranged from 0 (indicating poor agreement) to 0.82 (indicating substantial agreement) [17]. The domains consistently having the lowest ratings of importance and least agreement were "economic factors" (average importance across time point, disease-type and age group: 5.42 ± 2.85 [M ± SD]; average free marginal kappa across time point, disease-type and age group: -0.01) and "global treatment satisfaction" (average importance across timepoint, disease-type and age group: 6.83 ± 2.35 [M ± SD]; average free marginal kappa across timepoint, disease-type and age group: 0.24).

**Table 1:** Ratings of pain domain importance by diagnosis and age groupings

	Arthritis		Idiopathic musculoskeletal pain		Other rheumatic diseases		Average	Difference by point of assessment?
			
	Younger	Older	Younger	Older	Younger	Older		
Pain intensity	7.47 ± 2.48	8.25* ± 1.89	7.69 ± 2.19	8.26* ± 1.90	7.32 ± 2.30	7.96* ± 1.85	7.89 ± 1.76	No
Global rating of treatment satisfaction	6.53 ± 2.70	7.22* ± 2.47	6.41 ± 2.48	6.97* ± 2.39	6.57 ± 2.59	7.12* ± 2.43	6.83 ± 2.35	Follow-up > initial
Symptoms and adverse events	7.91 ± 2.36	8.09^† ^± 2.25	7.47 ± 2.50	7.76* ± 2.35	7.89 ± 2.27	8.07^† ^± 2.15	7.91 ± 2.19	No
Physical functioning	8.89 ± 1.65	8.97 ± 1.40	8.78 ± 1.64	8.86 ± 1.50	8.77 ± 1.60	8.83 ± 1.45	8.86 ± 1.42	No
Emotional functioning	7.52 ± 2.27	8.13* ± 1.76	8.03 ± 2.05	8.44* ± 1.68	7.71 ± 2.04	8.08* ± 1.78	8.01 ± 1.75	No
Role functioning	6.95 ± 2.45	8.00* ± 1.78	7.41 ± 2.34	8.21* ± 1.83	7.26 ± 2.28	8.04* ± 1.77	7.64 ± 1.86	No
Sleep	7.36 ± 2.13	7.54^† ^± 1.95	7.88 ± 2.01	8.31* ± 1.81	7.42 ± 2.06	7.59^† ^± 1.91	7.69 ± 1.76	No
Economic Factors	5.46 ± 2.96	5.55 ± 2.90	5.22 ± 2.91	5.33^† ^± 2.87	5.48 ± 2.97	5.51^† ^± 2.91	5.42 ± 2.85	No
**Average**	7.20 ± 1.78	7.67* ± 1.52	7.29 ± 1.63	7.73* ± 1.41	7.20 ± 1.66	7.58* ± 1.49	7.55 ± 1.44	

All values are M ± SD on a 0-10 metric; *, significant difference by age at p < 0.01; ^†^, significant difference by age at p < 0.05

Recommendations for additional areas to evaluate based on open-ended responses were classified into domains similar to PedIMMPACT (see Documents, Additional file 1 Digital Content 1). Based on the results of the first iterative survey, all PedIMMPACT domains other than "economic factors" were retained for possible inclusion in the final assessment tool. Potential specific items to assess within each general domain, based on open-ended responses, also were retained for rating in a second iterative survey. Given that importance ratings generally did not significantly differ by time point (initial versus follow-up assessment), no time point distinction was made in the second iterative survey.

#### Delphi Survey - Iteration 2

Of the 251 CARRA members sent requests for study participation, 157 (63%) completed the survey. The respondent group was made up of 83 Iteration 1 participants (73% of the initial respondents) plus additional CARRA members who had not completed the Iteration 1 survey. Participants were predominantly female (63%) and physicians (95%), with fellows comprising 20% of the physician group. Both average ratings and level of agreement were used to determine items for retention in this survey iteration. For level of agreement, responses were classified into minimal importance (0-3), moderate importance (4-6), and significant importance (7-10) and examined for agreement using multi-rater free-marginal kappa [16]. Items having an average rating of at least 7/10 for importance with a free-marginal kappa coefficient of at least 0.30 (indicating at least fair agreement on categorizing importance) [17] were retained.

The item with the highest average importance rating was the impact of pain on activities of daily living (ADL) (M ± SD: 9.04 ± 1.74); the item with the lowest average importance rating was pain unpleasantness (M ± SD: 5.19 ± 2.69). Low levels of agreement for importance were found for pain unpleasantness (κ = 0.00), sensory descriptors of pain (κ = 0.02), diet (κ = 0.05), comfort goal (κ = 0.06), and alcohol/drug use (κ = 0.11). The highest levels of agreement for importance were found for pain duration (κ = 0.53), stiffness (κ = 0.53), pain impact on physical activity (κ = 0.55), pain impact on school performance (κ = 0.79), and pain impact on ADL (κ = 0.83). The majority of participants (ranging from 56% to 93%) reported that their importance ratings would not significantly differ as a function of age or condition (see Documents, Additional file 2 Digital Content 2 for descriptive statistics for importance ratings by item).

#### Consensus Conference

Consensus conference discussion yielded general agreement on the following domains and items for inclusion: pain characteristics (current pain, average pain intensity over past 2 weeks, pain episode duration, pain frequency, pain location), associated symptoms (fatigue frequency), cognitive and emotional factors (catastrophizing, positive affect, sadness, anger, worry, stressors), and functioning (physical, social, and role). Areas agreed to be omitted included: pain sensory descriptors, pain aggravating/alleviating factors, pain unpleasantness, comfort goal, global pain treatment satisfaction rating, fatigue, appetite, pain self-efficacy, recent peer group changes or conflicts, and level of independence. During the consensus conference, lack of agreement over the specific questions and metrics to use for quantifying items (except for pain intensity) resulted in the assignment of workgroups to facilitate discussion and provide recommendations thereafter. Items for the functioning domain were obtained from the PROMIS pediatric pain interference scale [18]. Items for assessing the recommended cognitive dimension of pain (pain catastrophizing) were taken from the three highest loading items on the Pain Catastrophizing Scale for Children [19]. Pain location was captured using a body outline by von Baeyer and colleagues [20]. Remaining items were developed by investigators following literature review. The final measures included self-report measures for children (4 to 7 years) and youth (8 to 18 years) and a proxy-report for parents of children between 4 and 7 years (see Documents, Additional files Digital Content 3, 4, 5 for the final developmentally-appropriate SUPER-KIDZ measure for children, youth and parents).

### Phase 2: Feasibility Testing of Three Methods of Administering the Pain Measures

#### Demographic and disease characteristics of child and youth participants

Seventy-seven youth and 24 children (and their parents) participated in the study across the 4 sites. Of the children, 20 participants were included the analyses as the parents of 4 children inadvertently completed the child self-report portion of the assessment. Demographic and disease characteristics for the child and youth participants are summarized in Table 2. Youth reported current pain as 3.0 ± 2.1/10 (M ± SD) and children reported 1.4 ± 2.3/10 (M ± SD). These scores are derived from those recorded on the SUPER-KIDZ pain tool *via *the computer. Youth assessed their pain intensity using an 11-point (0-10) numerical rating scale and children assessed their pain intensity using the Faces Pain Scale-Revised [21] (see Documents, Additional files Digital Content 3, 4, 5). There was no difference in current pain intensity reported on different mediums for either group (youth: p = 0.652; children: p = 0.094).

**Table 2:** Demographic and disease characteristics of child and youth participants

	Children aged 4-7 years (n = 20)		Youth aged 8-18 years (n = 77)	
*Characteristic*	*M ± SD*	*n (%)*	*M ± SD*	*n (%)*
Age (years)	5.9 ± 0.9		13.5 ± 3.1	
Gender				
Female		12 (60.0)		49 (63.6)
Male		8 (40.0)		28 (36.4)
Primary Diagnosis				
Juvenile idiopathic arthritis				
Enthesitis related		0 (0.0)		10 (13.0)
Oligoarthritis		3 (15.0)		6 (7.8)
Polyarthritis (RF negative)		3 (15.0)		13 (16.9)
Polyarthritis (RF positive)		1 (5.0)		4 (5.2)
Psoriatic		1 (5.0)		3 (3.9)
Systemic		0 (0.0)		6 (7.8)
Unknown subtype		3 (15.0)		3 (3.9)
Juvenile dermatomyositis		2 (10.0)		5 (6.5)
Systemic lupus erythematosus		0 (0.0)		4 (5.2)
Other		2 (10.0)		16 (20.8)
Not yet diagnosed		1 (5.0)		7 (9.1)
Visit type				
Follow-up		17 (85.0)		63 (81.8)
New patient		3 (15.0)		14 (18.2)
Duration of pain (years)	2.1 ± 1.6^a^		5.3 ± 4.3	
Physicians disease severity rating (10 cm VAS)	2.1 ± 2.1^b^		2.4 ± 1.8^c^	

^a^, data are for n = 19 children; ^b^, data are for n = 14 children; ^c^, data are for n = 44 youth; RF = rheumatoid factor; VAS = visual analogue scale.

#### Outcome measures

Primary outcome measures for feasibility testing included: efficiency, missed responses, and participant acceptability. Efficiency was defined as time (in minutes) taken to complete the questionnaire. Missed responses were defined as any omission or incomplete question, regardless of the reason for omission. Reasons for missed responses included participant error and technical difficulty. Missed response data are displayed as the percentage of questions missed when completing the survey by a given medium. Only questions that had discrete response possibilities were included. Therefore, the pain body map question was not included in the missed response analysis, as any number of selectable body parts could have been chosen by a child or parent thus making it impossible to differentiate between omissions and purposeful non-selections. Acceptability described a participant's likes and dislikes related to a given medium.

#### Efficiency

Variances existed in the amount of time required to complete the questionnaire by medium for youth (p = 0.001) and parents (p = 0.001) as shown in Table 3. In each of these groups, completing the survey using the handheld device took the greatest amount of time (5.90 ± 2.79 minutes for 8-18 year olds and 7.00 ± 4.08 minutes for parents). There was no significant difference in mean efficiency between mediums when completing the 2-item survey used by children. With this group, the time to completion *via *each medium ranged from 1.64 ± 1.50 and 1.91 ± 1.81 minutes.

**Table 3:** Efficiency and missed responses on age-appropriate SUPER-KIDZ pain measure for paper, computer and handheld mediums

Measure	n	Paper Medium		Computer Medium		Handheld Medium		*P*-value^a^
			
		Mean ± SD	95% CI	Mean ± SD	95% CI	Mean ± SD	95% CI	
Efficiency (minutes)								
*Youth (8-18 years)*	62	3.08 ± 1.66	2.67, 3.49	3.40 ± 1.53	3.02, 3.78	5.90 ± 2.79	5.21, 6.60	0.001
*Children (4-7 years)*	11	1.91 ± 1.81	0.84, 2.98	1.64 ± 1.50	0.75, 2.52	1.82 ± 1.17	1.13, 2.51	0.638
*Parents of children*	14	2.28 ± 1.32	1.59, 2.98	4.00 ± 1.71	3.10, 4.90	7.00 ± 4.08	4.86, 9.14	0.001
Missed responses (percentage of total discrete questions asked^b^)								
*Youth (8-18 years)*	77	1.16 ± 3.48	0.38, 1.94	0.14 ± 0.84	-0.05, 0.32	3.42 ± 16.24	-0.21, 7.04	0.047
*Children (4-7 years)*	19	0.00 ± 0.00		0.00 ± 0.00		5.26 ± 22.94	-5.05, 15.58	0.368
*Parents of children*	23	1.90 ± 4.39	0.11, 3.70	0.54 ± 2.61	-0.52, 1.61	0.82 ± 2.86	-0.35, 1.98	0.156

^a^, for between medium comparison, ^b ^body map question not included in analysis

#### Missed Responses

Overall, the incidence of missed responses in all participant groups was low (Table 3). A significant difference in the number of missed responses between mediums was seen for youth (p = 0.047). Within this group, the largest percentage of missed responses occurred when using the handheld device. This phenomenon was largely driven by technical difficulties as the responses for 2 youth were not saved in the database despite survey completion. This resulted in 100% missed response rates for these participants. There were no significant differences in the number of missed responses between mediums for parents or children.

#### Acceptability

There was no difference in the overall preferred medium for youth or parents. There was however a significant difference in the preferred medium for children (p = 0.008) with 65% (n = 13) of parents reported their child preferred using the computer because the computer was the most simple and fun to use. A significant difference in medium least preferred existed only for youth (p = 0.001). Here 54% (n = 42) of youth disliked the handheld device citing its small size, unfamiliarity to the user and lengthy time to register responses. Not surprisingly, given the efficiency data, the computer or paper assessments were perceived to be quicker than the handheld device by the majority of youth (87%; n = 67; p = 0.001) and parents (91%; n = 21; p = 0.019). The majority of parents (91%; n = 21) also found the computer or paper to be easier to understand than the handheld device (p = 0.032) and 78% (n = 60) of youth found the coxmputer or paper more useful for describing pain than the handheld device (p = 0.027). There was also a significant difference in the medium youth felt was most appropriate for their age group (p = 0.004) with only 16% (n = 12) citing the paper medium as meeting this criterion.

#### Physician demographics and satisfaction

Fifteen physicians (73% female; 67% practicing longer than 10 years) participated in the study. A statistically significant difference existed in the method of data review thought to be the most time efficient (p = 0.022) with 67% (n = 10) of pediatric rheumatologists finding the concise computer-based summary the quickest to review. The majority of rheumatologists (67%; n = 10) also indicated no preference between the computer summary and paper questionnaire for developing pain management plans. There were no other significant differences in the method found most acceptable to physicians, however, the majority of physicians (60%; n = 9) would recommend the computer-generated summary to a colleague.

#### Missing data and technical problems

Although 101 youth and children (with parents) participated in the study, not all analyses include data from each participant. This is a result of data recording errors by RAs (e.g. collection of physician severity ratings), missing to measure time to survey completion by RAs and missed responses by participants on satisfaction surveys. In all cases, demographic and feasibility data presented are shown with the number of participants included in that analysis. During feasibility testing, we also experienced technical problems associated with the use of the handheld device and computer resulting from weak wireless network signals and leading to extended times to register responses and missed registration of entered responses.

## Discussion

We present the development of a standardized measure, SUPER-KIDZ, to assess pain in children and youth presenting to rheumatology practices. This measure is the product of a validated iterative process that consisted of a 2-stage Delphi survey followed by a consensus conference using nominal group technique. The self- and proxy-report measures were evaluated for feasibility of administration using paper, laptop and handheld-based mediums. We found that either paper or computer-based administration of the SUPER-KIDZ pain measures is clinically feasible and acceptable to patients and health care providers.

CARRA members generally agreed with the larger pediatric pain literature (and specifically PedIMMPACT recommendations on important pain dimensions to assess in practice. In particular, characteristics of pain (intensity, location, frequency, and duration), known pain modifiers (cognitive/affective variables) and consequences (functional limitations) were identified as most highly important to assess in pediatric rheumatology practice. This was generally irrespective of diagnosis or type of visit (initial versus follow-up). Attending to these aspects of a child's pain presentation is known to be instrumental for identifying patients at risk of enduring physical and/or emotional disability, selecting pain interventions, and monitoring treatment response [1]. However, the multidimensional assessment of pain if often omitted or inconsistently applied in pediatric rheumatology practices due to various perceived barriers (e.g., time) despite pain assessment being regarded as a quality care indicator for pediatric rheumatology [3]. It is hoped that the brief consensus pain measure developed during the present study ultimately will be helpful for ensuring quality care in pediatric rheumatology practice while providing clinician-friendly data on what is viewed as important aspects of a child's pain experience.

In order to determine a clinically feasible method for routinely implementing the SUPER-KIDZ measure in pediatric rheumatology practice, the present study compared three different mediums for assessment delivery (paper, computer, and handheld device). In general, electronic approaches to self-report assessment previously have been found to be well-regarded by pediatric and adult respondents and to have improved validity over paper-based methods [22-24]. Use of electronic assessment methods also naturally integrate with other forms of health information technology (e.g., electronic health records) and thus fit within the vision for contemporary pediatric medicine [25]. Results of the present study suggested that although use of handheld devices or computers had advantages for pain assessment, there were potential problems as well. In particular, use of a handheld device was perceived as burdensome to some participants due to a small screen size, technical problems, and complexity. Further, data recorded on the handheld device occasionally were not wirelessly transferred to an online database as intended, resulting in missing data, which could be problematic in practice. In addition, although rheumatologists preferred viewing computer-based pain assessment reports in general, they also responded favorably to viewing raw answers on a paper-based form. Thus, the optimal medium for implementing pain assessment in pediatric rheumatology practice may vary depending on the technology available and provider preference.

Our study has several limitations that may temper interpretation of results and conclusions. During Phase 1, the response rate of CARRA members varied from 46-63% and therefore study data may not be fully representative of all members. Despite using standardized consensus methodologies to establish a comprehensive but brief measure, important items may have been left off the final instrument that may limit the usefulness of the tool in certain contexts. Furthermore, SUPER-KIDZ was tested for feasibility only; evaluation of predictive utility and responsiveness is required before wide scale implementation can be recommended. Future studies are planned with this focus.

## Conclusions

It is feasible to implement a standardized, brief but multidimensional pain assessment (SUPER-KIDZ) using electronic or paper administration in routine pediatric rheumatology practice. In general, the computer-based administration of SUPER-KIDZ was most efficient, but the medium of pain assessment employed in practice may depend on age of the child, available resources and other administrative factors. Future research evaluating the reliability and predictive validity of the pain assessment tool is planned and is necessary prior to widespread implementation. Ultimately, it is hoped that the SUPER-KIDZ pain assessment tool will assist pediatric rheumatologists and allied health care professionals to use more consistent approaches to pain assessment such that pain management interventions and monitoring of pain outcomes in children and youth with rheumatic disease are more uniform across practices. In addition, a simple, comprehensive, and validated method to assess pain will facilitate inclusion as a critical outcome in clinical trials of rheumatic diseases of childhood.

## Abbreviations

ACR: American College of Rheumatology; CARRA: Childhood Arthritis and Rheumatology Research Alliance; PedIMMPACT: Pediatric Initiative on Methods, Measurement, and Pain Assessment in Clinical Trials; PRINTO: Pediatric Rheumatology International Trials Organization; PRCSG Pediatric: Rheumatology Collaborative Study Group; PROMIS: Patient Reported Outcomes Measurement Information System; RA: Research assistant; SPSS, Statistical Package for the Social Sciences; SUPER-KIDZ: Standardized Universal Pain Evaluation for pediatric rheumatology providers.

## Competing interests

The authors declare that they have no competing interests.

## Authors' contributions

JS and MC were responsible for study conception, participant recruitment, data interpretation and analysis and manuscript drafting. LJ was responsible for participant recruitment, data analysis and manuscript drafting. LES, GW, LRS, ST were responsible for participant recruitment. JS, MC, LES, GW, LRS, ST, ES, PC and MR were involved in tool creation. All authors were involved in critical manuscript revision for important intellectual content and gave their final approval of the manuscript.

## Supplementary Material

Additional file 1 **Summary of sample open-ended response items for SUPER-KIDZ pain measure**. Tabled summary of sample open-ended response items voted on for inclusion in the SUPER-KIDZ pain measure. Click here for file

Additional file 2 **Ratings of item importance from the second iterative survey**. Tabled summary of ratings of item importance from the second iterative survey. Click here for file

Additional file 3 **SUPER-KIDZ Pain Self-Report Tool (Ages 4-7)**. Visual presentation of tool discussed in manuscript. Click here for file

Additional file 4 **SUPER-KIDZ Pain Self-Report Tool (Ages 8-18)**. Visual presentation of tool discussed in manuscript. Click here for file

Additional file 5 **SUPER-KIDZ Pain Self-Report Tool (Parent Proxy for Ages 4-7)**. Visual presentation of tool discussed in manuscript. Click here for file
