# Supplementary material for: Developing a standardized approach to the assessment of pain in children and youth presenting to pediatric rheumatology providers: a Delphi survey and consensus conference process followed by feasibility testing
Source: Pediatr Rheumatol Online J. 2012 Apr 10;10:7. doi: 10.1186/1546-0096-10-7 (PMC3366881; doi:10.1186/1546-0096-10-7)
Supplement: Additional file 1 — Summary of sample open-ended response items for SUPER-KIDZ pain measure. Tabled summary of sample open-ended response items voted on for inclusion in the SUPER-KIDZ pain measure. [file 1546-0096-10-7-S1.PDF]

SUPER-KIDZ

Recommended Self-Report Version for Children Aged <8 Years

(1) These faces show how much something can hurt. This first face shows no pain. The faces show more and more pain up to the last face – it shows very much pain. Click on the face that shows how much you hurt right now.

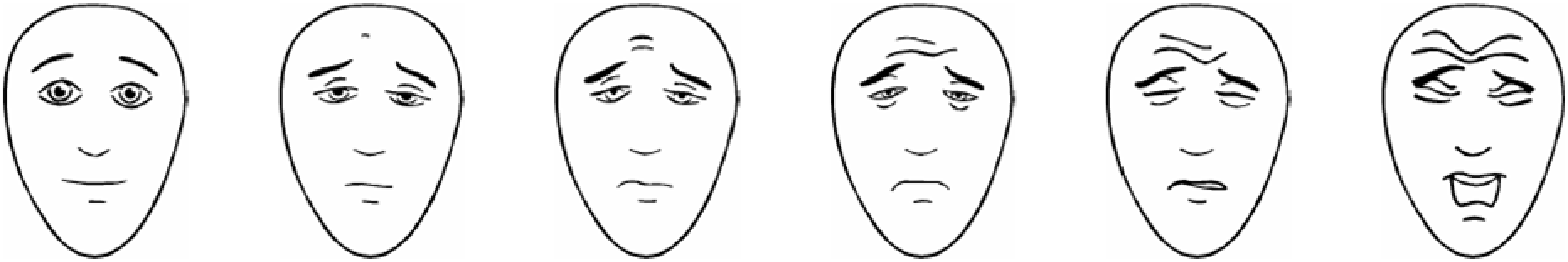

(2) Click on all the parts of your body where you have had pain in the past 7 days.

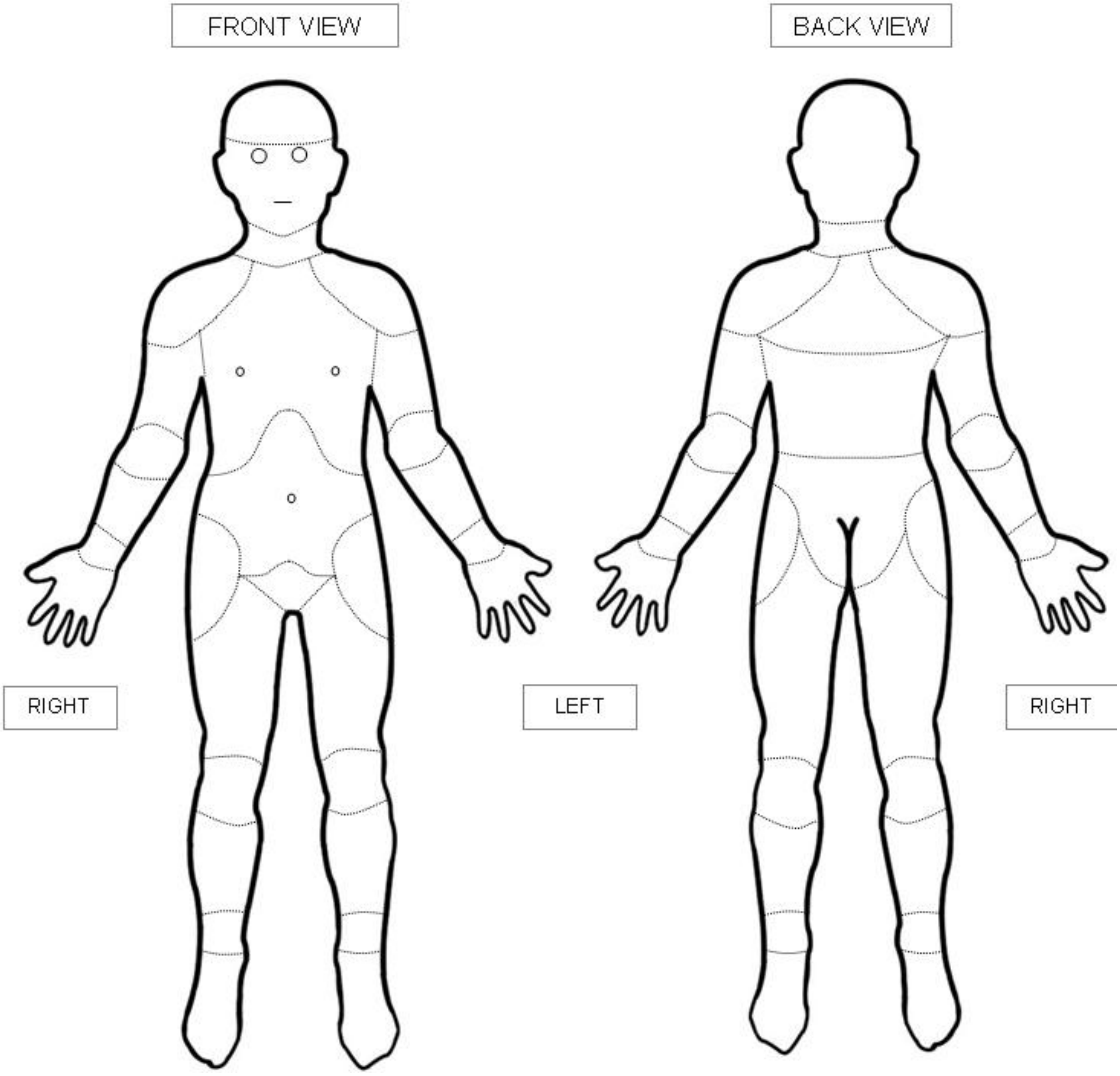

Thank you for answering these questions.
