# Supplementary material for: Developing a standardized approach to the assessment of pain in children and youth presenting to pediatric rheumatology providers: a Delphi survey and consensus conference process followed by feasibility testing
Source: Pediatr Rheumatol Online J. 2012 Apr 10;10:7. doi: 10.1186/1546-0096-10-7 (PMC3366881; doi:10.1186/1546-0096-10-7)
Supplement: Additional file 2 — Ratings of item importance from the second iterative survey. Tabled summary of ratings of item importance from the second iterative survey. [file 1546-0096-10-7-S2.PDF]

SUPER-KIDZ

Recommended Self-Report Version for Children Aged >=8 Years

(1) How much pain do you have *right now*? Check the box below the number that best describes your level of pain right now, with 0 being "no pain" and 10 being "most pain possible."

No pain	0	1	2	3	4	5	6	7	8	9	10	Most pain possible
	☐	☐	☐	☐	☐	☐	☐	☐	☐	☐	☐	

(2) If you had pain *in the past 7 days,* how much did it usually hurt? Check the box below the number that best describes your usual level of pain during *the past 7 days,* with 0 being "no pain" and 10 being "most pain possible."

No pain	0	1	2	3	4	5	6	7	8	9	10	Most pain possible
	☐	☐	☐	☐	☐	☐	☐	☐	☐	☐	☐	

(3) On how many days did you have pain *in the past 7 days?*

☐ 1 day
☐ 2 days
☐ 3 days
☐ 4 days
☐ 5 days
☐ 6 days
☐ Every day

(4) If you had pain *in the past 7 days*, how long did the pain usually last?

☐ Less than 30 minutes
☐ About 1 hour
☐ Between 1 and 3 hours
☐ About half the day
☐ All day or longer
☐ No pain in the past 7 days

(5) Select all the parts of your body where you have had pain *in the past 7 days.*

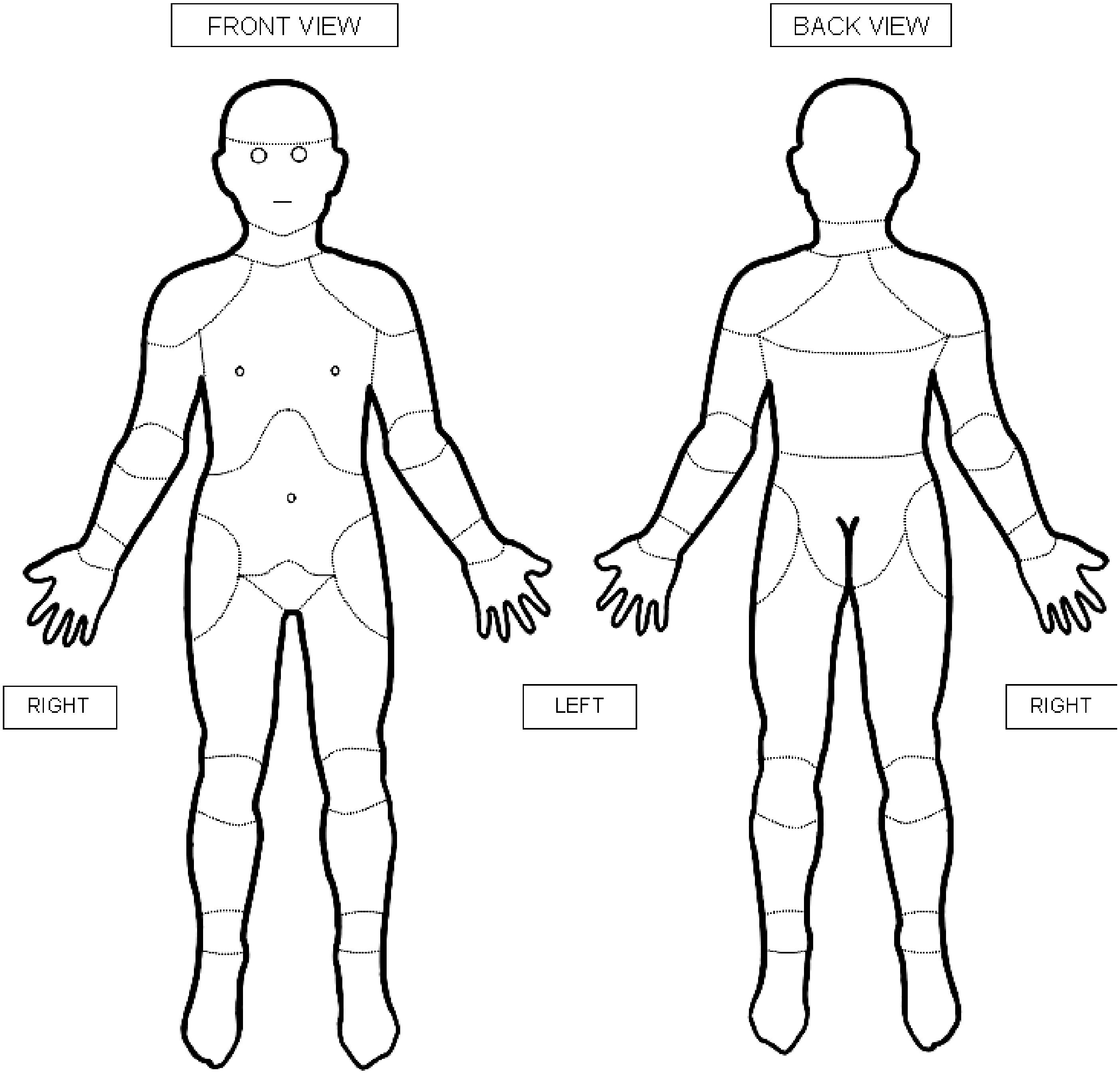

(6) *In the past 7 days,* how often have you felt tired?

- ☐ Never
- ☐ Almost never
- ☐ Sometimes
- ☐ Often
- ☐ Almost always

There are many possible ways that pain can affect lives of young people. Please respond to each item by checking one box per row.

In the past 7 days…

	Never	Almost Never	Sometimes	Often	Almost Always
(7) I had trouble sleeping when I had pain.	☐	☐	☐	☐	☐
(8) It was hard for me to pay attention when I had pain.	☐	☐	☐	☐	☐
(9) It was hard to stay standing when I had pain.	☐	☐	☐	☐	☐
(10) It was hard to have fun when I had pain.	☐	☐	☐	☐	☐
(11) I had trouble doing schoolwork when I had pain.	☐	☐	☐	☐	☐
(12) It was hard for me to walk one block when I had pain.	☐	☐	☐	☐	☐
(13) It was hard for me to run when I had pain.	☐	☐	☐	☐	☐
(14) I kept thinking about how much I wanted the pain to stop when I had pain.	☐	☐	☐	☐	☐
(15) I was afraid that the pain would get worse when I had pain.	☐	☐	☐	☐	☐
(16) I felt I couldn’t stand it anymore when I had pain.	☐	☐	☐	☐	☐

Below are some words that describe different feelings and emotions. Read each item, and then check one box per row under the word that describes how often you felt this way *in the past 7 days*.

	**Never**	**Almost Never**	**Sometimes**	**Often**	**Almost Always**
(17) Sad	☐	☐	☐	☐	☐
(18) Angry	☐	☐	☐	☐	☐
(19) Cheerful	☐	☐	☐	☐	☐
(20) Worried	☐	☐	☐	☐	☐

Thank you for answering these questions.
