# Supplementary material for: Developing a standardized approach to the assessment of pain in children and youth presenting to pediatric rheumatology providers: a Delphi survey and consensus conference process followed by feasibility testing
Source: Pediatr Rheumatol Online J. 2012 Apr 10;10:7. doi: 10.1186/1546-0096-10-7 (PMC3366881; doi:10.1186/1546-0096-10-7)
Supplement: Additional file 3 — SUPER-KIDZ Pain Self-Report Tool (Ages 4-7). Visual presentation of tool discussed in manuscript. [file 1546-0096-10-7-S3.PDF]

SUPER-KIDZ

Recommended Parent-Report Version for Children Aged < 8 Years

Instructions: Please answer the following questions about your child's pain from *your* view.

(1) How much pain do you think your child has *right now*? Check the box below the number that best describes your child's level of pain right now, with 0 being "no pain" and 10 being "most pain possible."

	0	1	2	3	4	5	6	7	8	9	10	
**No pain**	☐	☐	☐	☐	☐	☐	☐	☐	☐	☐	☐	**Most pain possible**

(2) If your child had pain *in the past 7 days*, how much did it usually hurt? Check the box below the number that best describes your child's usual level of pain during *the past 7 days*, with 0 being "no pain" and 10 being "most pain possible."

	0	1	2	3	4	5	6	7	8	9	10	
**No pain**	☐	☐	☐	☐	☐	☐	☐	☐	☐	☐	☐	**Most pain possible**

(3) On how many days did your child have pain *in the past 7 days?*

☐ 1 day
☐ 2 days
☐ 3 days
☐ 4 days
☐ 5 days
☐ 6 days
☐ Every day

(4) If your child had pain *in the past 7 days*, how long did the pain usually last?

☐ Less than 30 minutes
☐ About 1 hour
☐ Between 1 and 3 hours
☐ About half the day
☐ All day or longer
☐ No pain in the past 7 days

(5) Click on all the parts of the body where your child has had pain *in the past 7 days.*

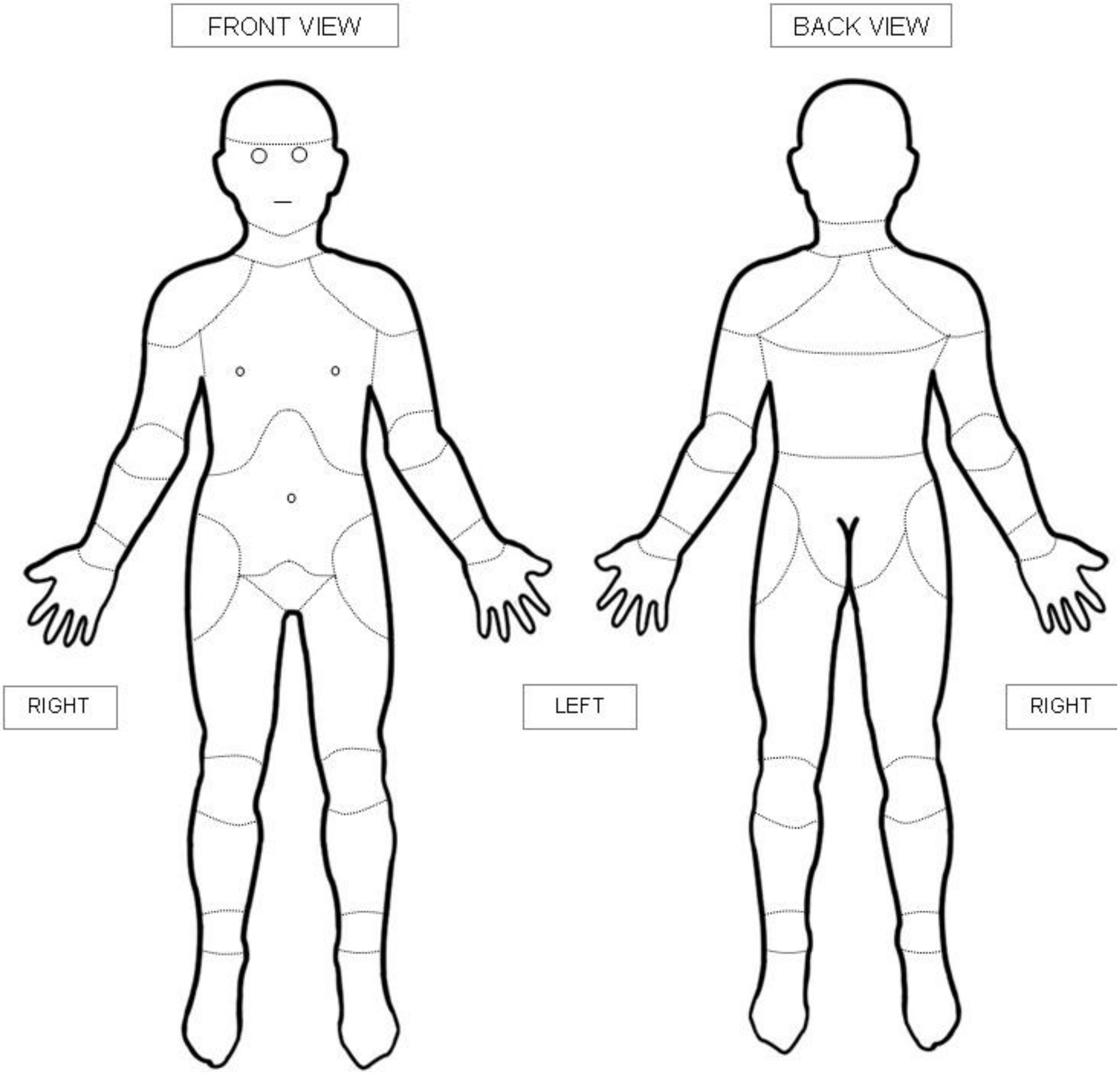

(6) *In the past 7 days,* how often has your child been tired?

- ☐ Never
- ☐ Almost never
- ☐ Sometimes
- ☐ Often
- ☐ Almost always

There are many possible ways that pain can affect lives of young people. Please respond to each item by checking one box per row.

In the past 7 days…

	Never	Almost Never	Sometimes	Often	Almost Always
(7) It was hard for my child to sleep when he/she had pain.	☐	☐	☐	☐	☐
(8) It was hard for my child to pay attention when he/she had pain.	☐	☐	☐	☐	☐
(9) It was hard for my child to stay standing when he/she had pain.	☐	☐	☐	☐	☐
(10) It was hard for my child to have fun when he/she had pain.	☐	☐	☐	☐	☐
(11) It was hard for my child to do schoolwork when he/she had pain.	☐	☐	☐	☐	☐
(12) It was hard for my child to walk one block when she/he had pain.	☐	☐	☐	☐	☐
(13) It was hard for my child to run when he/she had pain.	☐	☐	☐	☐	☐

Below are some words that describe different feelings and emotions. Read each item and then check the box under the word that describes how often your child has felt this way *in the past 7 days*.

	**Never**	**Almost Never**	**Sometimes**	**Often**	**Almost Always**
(14) Sad	☐	☐	☐	☐	☐
(15) Angry	☐	☐	☐	☐	☐
(16) Cheerful	☐	☐	☐	☐	☐
(17) Worried	☐	☐	☐	☐	☐

Thank you for answering these questions.
