# Supplementary material for: Developing a standardized approach to the assessment of pain in children and youth presenting to pediatric rheumatology providers: a Delphi survey and consensus conference process followed by feasibility testing
Source: Pediatr Rheumatol Online J. 2012 Apr 10;10:7. doi: 10.1186/1546-0096-10-7 (PMC3366881; doi:10.1186/1546-0096-10-7)
Supplement: Additional file 4 — SUPER-KIDZ Pain Self-Report Tool (Ages 8-18). Visual presentation of tool discussed in manuscript. [file 1546-0096-10-7-S4.PDF]

Supplemental Digital Content 1. Summary of sample open-ended response items for SUPER-KIDZ pain measure

Domain	Open-Ended Response Items
Pain intensity/characteristics	• Location of pain<br>• Sensory descriptors of pain<br>• Pain alleviating and aggravating factors<br>• Pain duration<br>• Pain frequency<br>• Pain unpleasantness<br>• Spontaneous versus evoked pain<br>• Tolerable level of pain intensity/comfort goal
Treatment satisfaction	• Effectiveness of pain treatments tried to date<br>• Global pain treatment satisfaction rating
Associated symptoms	• Stiffness<br>• Fatigue/energy level<br>• Disrupted sleep
Physical functioning	• Impact of pain on participation in sports and physical activities<br>• Impact of pain on activities of daily living
Emotional Functioning	• Impact of pain on mood (anxiety, depression)<br>• Pain coping<br>• Self-efficacy

	• Recent stressors/social changes
Role Functioning	• School absenteeism and performance<br>• Restrictions in play activities<br>• Involvement with peers<br>• Level of independence
Sleep/lifestyle factors	• Sleep habits<br>• Exercise<br>• Diet<br>• Use of alcohol, nicotine, or illicit substances
