# Supplementary material for: Developing a standardized approach to the assessment of pain in children and youth presenting to pediatric rheumatology providers: a Delphi survey and consensus conference process followed by feasibility testing
Source: Pediatr Rheumatol Online J. 2012 Apr 10;10:7. doi: 10.1186/1546-0096-10-7 (PMC3366881; doi:10.1186/1546-0096-10-7)
Supplement: Additional file 5 — SUPER-KIDZ Pain Self-Report Tool (Parent Proxy for Ages 4-7). Visual presentation of tool discussed in manuscript. [file 1546-0096-10-7-S5.PDF]

Supplemental Digital Content 2. Ratings of item importance from the second iterative survey

Item	Rating*	Not Important 0-3 (%)	Somewhat Important 4-6 (%)	Very Important 7-10 (%)
Pain unpleasantness	5.19±2.69	30.7	32.9	36.50
Sensory descriptors of pain	5.34±2.35	23.5	42.7	33.8
Diet	5.73±2.15	17.4	44.0	38.5
Comfort goal	5.97±2.73	18.1	33.8	48.1
Alcohol or drug use	6.42±2.47	12.7	34.6	52.7
Spontaneous versus evoked pain	6.45±2.13	9.5	37.2	53.3
Fatigue	6.47±2.45	11.4	35.1	53.5
Pain self-efficacy	6.59±2.35	13.2	28.1	58.8
Global pain treatment satisfaction	6.77±2.33	10.8	25.8	63.3
Involvement with peers	7.06±1.98	6.4	25.7	67.9
Effectiveness of pain treatment(s) tried	7.25±2.23	7.6	18.6	73.8
Level of independence	7.28±2.21	6.4	29.1	64.5
Pain coping	7.32±2.06	7.9	16.7	75.4

Exercise	7.40±2.05	6.4	18.4	75.2
Sleep habits	7.45±2.28	6.4	17.4	76.2
Sleep disruption	7.94±2.19	6.1	13.0	80.9
School performance	7.49±1.96	3.6	3.6	92.8
Alleviating and aggravating factors	7.49±1.97	5.1	19.7	75.2
Recent stressors	7.49±2.07	5.3	21.0	73.7
Pain impact on mood	7.57±2.09	4.4	21.0	74.6
Pain location	7.63±2.45	8.8	21.2	70.0
Frequency of pain	7.71±1.95	2.9	22.6	74.5
Pain intensity	7.89±2.47	8.8	16.1	75.1
Restrictions in play activities	7.92±1.94	3.6	17.1	79.3
Pain duration	7.92±1.77	1.5	17.5	81.0
Stiffness	7.97±2.14	5.2	13.0	81.8
Pain impact on physical activity	7.99±1.79	2.6	14.9	82.5
Pain impact on activities of daily living	9.04±1.74	2.6	3.5	93.9

*, values are M±SD on a 0-10 metric.
